# Dysregulated Exosomes Result in Suppression of the Immune Response of Pregnant COVID-19 Convalescent Women

**DOI:** 10.3389/fmolb.2022.869192

**Published:** 2022-05-13

**Authors:** Hang Cao, Nor Haslinda Abd Aziz, Janet Raja Xavier, Mohamad Nasir Shafiee, Aida Kalok, Babban Jee, Madhuri S. Salker, Yogesh Singh

**Affiliations:** ^1^ Department of Women’s Health, Research Institute for Women’s Health, University of Tübingen, Tübingen, Germany; ^2^ Department of Obstetrics and Gynaecology, Faculty of Medicine, Universiti Kebangsaan Malaysia, Kuala Lumpur, Malaysia; ^3^ Department of Health Research, Ministry of Health and Family Welfare, Government of India, New Delhi, India; ^4^ Institute of Medical Genetics and Applied Genomics, University of Tübingen, Tübingen, Germany; ^5^ NGS Competence Centre Tübingen (NCCT), University of Tübingen, Tübingen, Germany

**Keywords:** pregnancy, COVID-19, exosomes, inflammation, PBMC

## Abstract

A successful pregnancy outcome is dependent on a delicate balance between inflammatory and anti-inflammatory processes throughout the different trimesters. Interruption in this balance can lead to an adverse outcome resulting in pregnancy loss. Since late 2019, the emergence of the new SARS-CoV-2 virus has affected lives worldwide, including pregnant women; therefore, there is an urgent need to address different approaches in relation to prevention, diagnostics, and therapeutics. Early pregnancy is affected by SARS-CoV-2 infection leading to fetal demise. Available evidence also suggests that 90% of pregnant women infected with the SARS-CoV-2 virus seem to be asymptomatic. Nonetheless, it is still unclear how COVID-19 affects exosome production in pregnant women recovered from COVID-19 and how these exosomes regulate the adaptive immune response. In this study, we found several exosomes including CD9, CD31, CD40, CD45, CD41b, CD42a, CD62P, CD69, CD81, CD105, and HLA-DRDPDQ in the plasma of COVID-19-recovered pregnant women were significantly less abundant than the control group. Furthermore, to understand how these exosomes affect the adaptive immune response, we co-cultured the peripheral blood mononuclear cells (PBMCs) from healthy control (HC) pregnant women with exosomes of either Preg-HC or Preg-recovered COVID-19 women. We identified that Preg-recovered COVID-19 women have reduced capacity for the inflammatory cytokine TNF-α from cytotoxic CD8^+^ T cells. In summary, our study highlights that pregnant recovered COVID-19 women have reduced production of several exosomes and possess fewer immunogenic properties. Our study implicates that exosomes can control inflammation and antigen presentation capacity of immune cells, thus limiting the infection in pregnant women.

## Introduction

The COVID-19 pandemic is a worldwide challenge for all of us currently due to the emergence of different variant strains which need several different approaches to overcome in terms of prevention, diagnostics, and therapeutics (Keni et al., 2020; Zeyaullah et al., 2021). Small extracellular vesicles (SEVs) or exosomes are emerging as a new front runner around viral infections including SARS-CoV-2 (Borowiec et al., 2021). Exosomes are a subtype of extracellular vesicles, and they are spherical biological structures released mostly by eukaryotic cells. Exosomes are replication-independent as they lack a functional nucleus (Théry et al., 2018). Exosomes consist of a lipid bilayer of a diameter of up to 5,000 nm with a structure similar to viruses, which are released into the intercellular space by most types of eukaryotic cells, both in physiological and pathological states (Borowiec et al., 2021).

The coronavirus may be internalized into the host cells possibly by caveolin-1-dependent endocytosis (Owczarek et al., 2018). SARS-CoV-2 infection of host cells has resulted in increased production of circulating exosomes (Bansal et al., 2021). Moreover, the occurrence of SARS-CoV-2 in exosomal cargo supports the hypothesis that this virus might utilize these extracellular vesicles for its spread (Barberis et al., 2021). Interestingly, exosomes play a dual role in host–pathogen interaction; on the one hand, these manipulate the immune response of the host for intracellular dissemination of virus (Bello-Morales et al., 2020; Gurunathan et al., 2021), while on the other hand, exosomes function as a potent antiviral agents against SARS-CoV-2 (Hassanpour et al., 2020; Gurunathan et al., 2021). Hence, the suppression of exosome production may be one of the vital antiviral strategies that can be of immense therapeutic value. Although the mechanism of SARS-CoV-2 infection in pulmonary dysfunction appears to be well described (Baggen et al., 2021), how exosomes affect the immune response of pregnant women is still not well understood.

Pregnancy is a state in which dynamics of the immune response and anatomical and physiological parameters are paramount for a successful outcome, and any disturbance in this complicated process and gap in communication could lead to unsuccessful pregnancy outcomes, such as pregnancy failure, pre-eclampsia, and fetal demise (Aghaeepour et al., 2017; Bhatia and Chhabra, 2018; Abu-Raya et al., 2020; Kidszun et al., 2020; Chen et al., 2021). Several recent articles have described that pregnant women become more severely sick than nonpregnant women, while others suggested that SARS-CoV-2-infected pregnant women are mostly asymptomatic (Alberca et al., 2020; Allotey et al., 2020; Kucirka et al., 2020; Mullins et al., 2020; Shende et al., 2020; Celewicz et al., 2021; Nidhi, 2021; Villar et al., 2021). Nonetheless, mechanisms of these discordant findings in pregnant women are not clear yet.

To understand the mechanisms of how SARS-CoV-2 infection could possibly affect the pregnancy outcome, we proposed a hypothesis that exosomes released from pregnant women after SARS-CoV-2 viral infection could compromise the immune responses, leading to the development of varied clinical characteristics.

In this study, we describe that pregnant women (mostly mild and asymptomatic) who recovered from SARS-CoV-2 viral infection had a reduced abundance of many exosomes, and these exosomes are poor in eliciting the immune response which could help pregnant women to limit the SARS-CoV-2 viral infection.

## Results and Discussion

**FIGURE 1:**
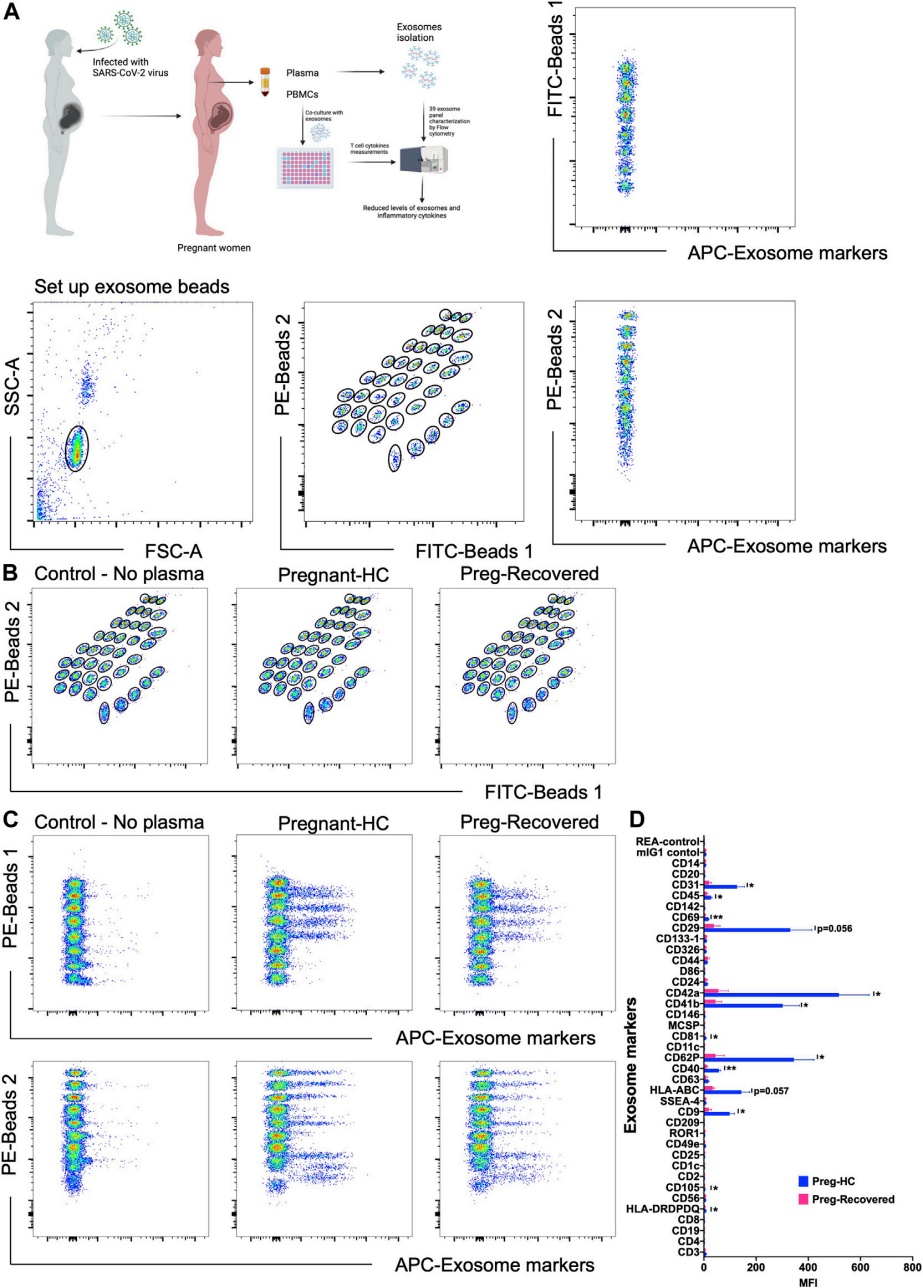
Detection of exosomal levels in COVID-19-recovered pregnant women. **(A)** Schematic picture showing the experimental strategy for the detection of exosome levels and *in vitro* immune response in PBMCs (left). The multiplex bead-based platform was used for the detection of exosome levels. Isolated exosomes were incubated for 1 h with 39 different bead populations. The different bead populations are distinguishable by flow cytometry. Exosomes bound to the beads were detected by using an equal volume of the exosome markers including 39 exosomal markers anti-CD9-APC, CD63-APC, and CD81-APC antibodies. The gating strategy applied for gating on single beads and showing exclusion of doublets and no bead events. The subsequent gates applied to identify all 39 capture beads show their fluorescence in the FITC *vs*. PE channel with adjunct dot plots showing respective APC-stained bead populations. **(B,C)** Gates applied to identify the 39 distinct capture bead populations by their fluorescence in the FITC *vs*. PE channel with adjunct dot plots showing respective APC-stained bead populations: Buffer control *vs*. pregnant healthy and control vs. pregnant COVID-19-recovered samples. **(D)** Representative quantification of the MFI values of exosome levels using specific exosome antibody-coated bead populations between pregnant healthy control (*n* = 7) and pregnant COVID-19-recovered samples (*n* = 3). p-value shows statistical significance among control and pregnant COVID-19-recovered samples, *p < 0.05, **p < 0.01.

**FIGURE 2:**
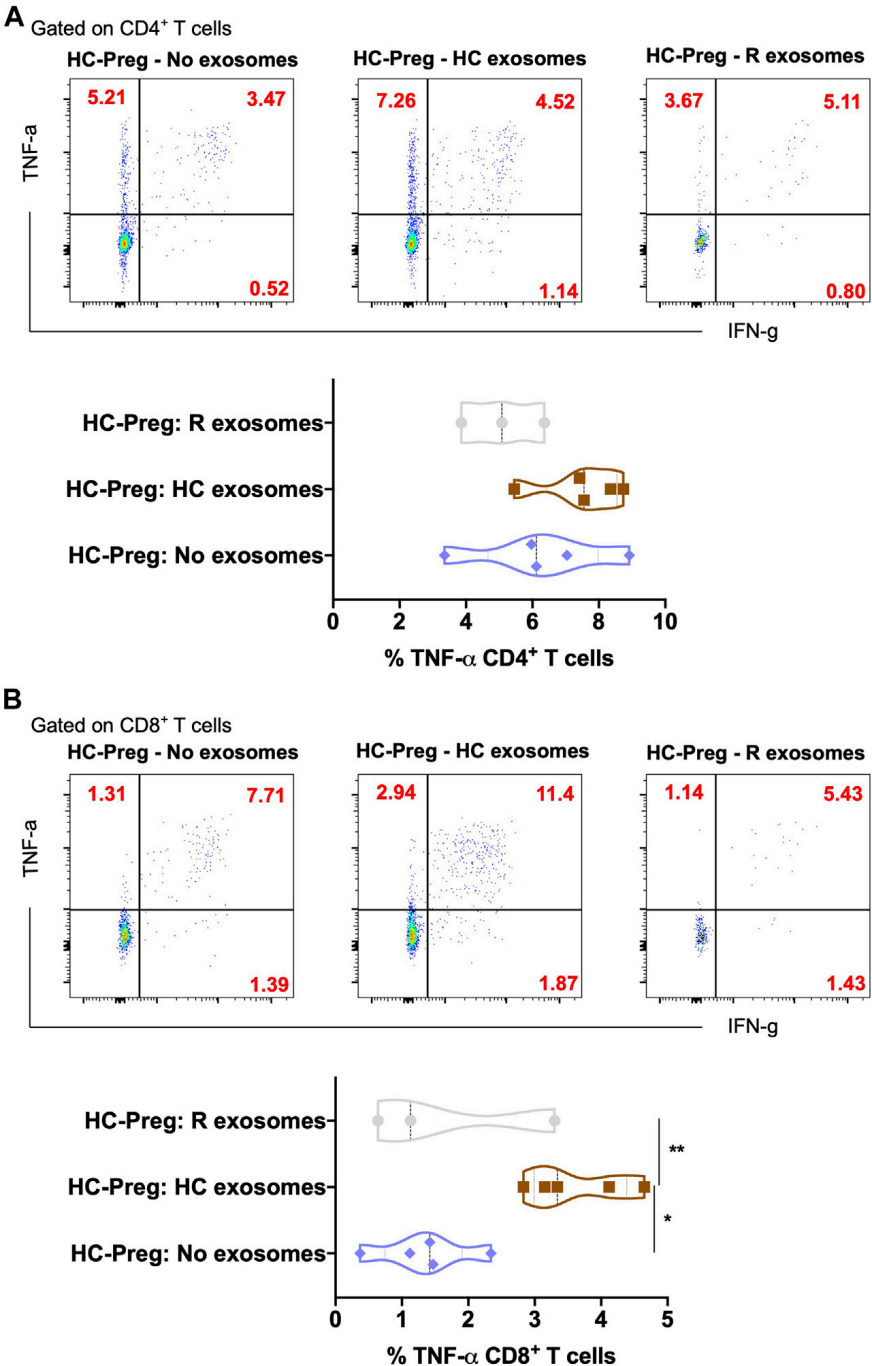
*In vitro* secretion of inflammatory cytokines in CD4^+^ T helper cells and CD8^+^ cytotoxic T cells upon exosome stimulation. Representative plots of TNF-α and IFN-γ in the CD4^+^ T cells **(A)** and CD8^+^ T cells **(B)** with the percentage of TNF-α upon the exosome stimulation from buffer control vs. pregnant healthy control vs. pregnant COVID-19-recovered sample. *n* = 3–5, p-value shows statistical significance among control and pregnant COVID-19-recovered samples, *p < 0.05, **p < 0.01.

Exosomes are mainly produced by virus-infected cells and mediate a proper intercellular communication among infected and uninfected cells (Gurunathan et al., 2021). SARS-CoV-2 modulates the production and composition of exosomes and can exploit exosome formation, secretion, and release pathways to promote its infection, transmission, and intercellular spread (Barberis et al., 2021). Therefore, we first characterized the 39 key exosomes using a bead-based exosome profiling kit in a total of 10 clinical samples (Figures 1A,B). We found that 11 of 39 exosomes [CD9 (*p* = 0.024), CD31 (*p* = 0.043), CD40 (*p* = 0.001), CD41b (*p* = 0.036), CD42a (*p* = 0.036), CD45 (*p* = 0.041), CD62P (*p* = 0.041), CD69 (*p* = 0.007), CD81 (*p* = 0.031), CD105 (*p* = 0.020), and HLA-DRDPDQ (*p* = 0.031)] were significantly less abundant (based on the equal amount of protein used for the detection of the exosomes in percentage) in pregnant recovered women (*n* = 3) than healthy control pregnant women (*n* = 7), while CD29 (*p* = 0.056) and HLA-ABC (*p* = 0.057) were nearly significant (Figures 1C,D). Most of the recovered women used in this study were asymptomatic during SARS-CoV-2 infection, as described in patient demographics (Table 1). Our data are in accordance with a recent finding in which it has been described that exosomal protein with biological processes such as complement activation, classical pathway, immune response, regulation of complement activation, Fc-gamma receptor signaling pathway involved in phagocytosis, and immunoglobulin production was downregulated in noncritical and critical COVID-19 patients compared with healthy controls (Barberis et al., 2021). These results support a similar pattern in our study that the COVID-19-recovered pregnant women who were asymptomatic also have less immunogenic exosome expression; thus, our pilot study strongly suggests that the host response to SARS-CoV-2 could be affected by exosomes. In our study, most of these exosomes appear to be involved in immune regulation pathways, antigen presentation, cell adhesion, cell activation, and co-stimulation process of innate and adaptive immune systems. The exosome markers CD29, CD31, CD41, CD42, and CD62P (cell adhesion and integrins), which are thought to support SARS-CoV-2 entry, are reduced in COVID-19-recovered pregnant women, so they could play a protective role during pregnancy to limit severe infection once pregnant women get infected. Previous studies suggested that exosomes are internalized into the recipient cells *via* several routes such as phagocytosis, macropinocytosis, endocytosis, and fusion (Gurunathan et al., 2021). Therefore, we took the healthy pregnant women’s peripheral blood mononuclear cells (PBMCs) and co-cultured with exosomes derived from either healthy control pregnant women (*n* = 5–7) or SARS-CoV-2-recovered pregnant women (*n* = 3). To our surprise, we found that exosomes derived from healthy control pregnant women elicit IFN-*γ* and TNF-α production from CD4^+^ T and CD8^+^ T cells, respectively (Figures 2A,B). However, exosomes derived from SARS-CoV-2-recovered pregnant women showed significantly reduced TNF-α (*p* = 0.038) production only from CD8^+^ T cells as compared to the healthy control (Figure 2B). We also checked the expression of other cytokines such as IL-2, IL-10, IL-17, and CCL2 (MCP-1) but could not find a significant difference between the two groups (data not shown). Our findings suggested that healthy noninfected pregnant women could have a strong immune response. If healthy pregnant women become infected during pregnancy with the SARS-CoV-2 virus, then a reduced exosome production may indeed interfere with a proper immune and cytokine pathway activation, thus leading to further entry of the virus into the body. In summary, despite having several limitations including small sample size and unavailability of pregnant women with severe COVID-19 for the study, we have shown that SARS-CoV-2-recovered pregnant women who were infected have a reduced amount of exosomal proteins and elicit an attenuated IFN-γ and TNF-α response from PBMCs to limit further aggravation/progression of the disease during pregnancy. This reduction may be crucial in limiting COVID-19 progression but not the infection since a higher level of these cytokines are an indication of cytokine storm that ultimately causes deterioration of the disease. However, further evidence is needed to understand the proper mechanisms of immune evasion.

**TABLE 1:** Patient demographics.

Study ID	Age (years)	Ethnicity	1st PCR test	Symptoms*	WHO score**	Sample collection date***	Gestational age (weeks)****
H2	35	Malay	—	—	—	24.11.20	31
H3	29	Malay	—	—	—	24.11.20	38
H7	31	Malay	—	—	—	14.12.20	35
H8	29	Malay	—	—	—	14.12.20	13
H9	37	Malay	—	—	—	15.12.20	37
H10	33	Malay	—	—	—	15.12.20	15
H11	27	Malay	—	—	—	16.12.20	38
M02 (R)	36	Malay	11.04.20	Symptomatic^#^	2	29.07.20	24
M05 (R)	36	Malay	11.04.20	Asymptomatic	1	13.04.20	36
M20 (R)	25	Malay	10.01.21	Symptomatic^§^	2	20.01.21	29

Symptoms*—symptoms at the time of disease development.

WHO score**—WHO score at the disease development (first PCR, test).

Gestational age****—gestational age during the blood (PBMCs) sample collection.

Symptomatic^#^—cough for 2 days.

Symptomatic^§^—fever for 2 days.

Limitations of the findings: A major limitation of the study is small sample size and unavailability of pregnant women with severe COVID-19 for the study during the sample collection from April 2020 to January 2021 in outpatient clinics (Malaysia). This is due to patients’ unwillingness to give samples for the research project, as this project was also a part of the study which involved the transcriptomics (genetic)-based identification/detection of COVID-19-specific markers in pregnant women. Furthermore, during this time, our group did not come across any pregnant women with severe COVID-19 in the outpatient clinics. Only three to four samples could be managed for study for approximately 10 months. This may be further impacted due to the reluctance of pregnant women to enter hospitals due to the possibility/risk of infection. Despite having had limited samples, we could observe a significant change in exosomes; thus, we believe the results are worthy of reporting. Nonetheless, our study opens future avenues of research and future implications of exosomes in the diagnostics and understanding of the cell-to-cell communication process during viral infection not only in pregnant women but also in other COVID-19 patients.

## Materials and Methods

### Ethics Statement for the Study Participants

This study is a part of the overall study of the transcriptomic and protein analyses of pregnant women with a history of COVID-19 infection at the epicenter of the COVID-19 pandemic in Malaysia approved by the Research Ethics Committee, National University of Malaysia (JEP-2021-465), and by the Medical Research Ethics Committee (MREC) of the Ministry of Health of Malaysia (ID-58736). This was a cross-sectional study carried out in Malaysia with a total of 10 pregnant women fulfilling the inclusion and exclusion criteria, who were selected using convenient sampling. In total, three pregnant women who had recovered from COVID-19 and seven pregnant women from healthy controls provided written informed consent to participate in this study (Table 1). Disease severity for this study was determined based on symptoms. These women were recruited from the obstetrics and gynecology clinic, outpatient clinic, and wards from April 2020 to January 2021. The study procedures were carried out in accordance with the Declaration of Helsinki.

### Exosomes Isolation Without Proteinase K Treatment

The serum samples were centrifuged at 2000 x*g* for 20 min at room temperature (RT), and the supernatant was then transferred to a new tube and subsequently centrifuged at 10,000 x*g* for 20 min. A volume of clarified serum was transferred to a new tube, and 0.5 volume of PBS was added, followed by 0.2 volume of Exosome Precipitation Reagent (#4484450, Thermo Fisher Scientific), and then vortexed. The homogeneous sample (which should have a cloudy appearance) was incubated at room temperature for 10 min. After incubation, the samples were centrifuged at 10,000 x*g* for 5 min at RT, and 0.5 volume PBS was added to the tube and vortexed to resuspend the exosomes. The isolated exosomes were kept at −20°C until use.

### Bead-Based Multiplex Exosome Flow Cytometry Assay

Different sample types were subjected to bead-based multiplex EV analysis by flow cytometry. All treatments were performed as per the product instructions (MACSPlex Exosome Kit, human, #130-108-813, Miltenyi Biotec). In brief, 20 µg EV-containing samples were diluted with MACSPlex buffer (MPB) to a final volume of 120 µl and loaded into 1.5-ml Eppendorf tubes before 15 µl of MACSPlex Exosome Capture Beads were added into each sample. Then, 5 µl of each APC-conjugated 39 antibodies such as anti-CD9, anti-CD63, and anti-CD81 (Miltenyi Biotec) detection antibodies were added to each tube and incubated on an orbital shaker at 450 rpm protected from light for 1 h at RT. Next, the samples were washed by adding 400 µl MPB to each tube by centrifugation at RT at 3000 x*g* for 5 min. This was followed by another washing step with 400 µl of MPB and again incubation on an orbital shaker at 450 rpm protected from light for 15 min at RT. Subsequently, the samples were resuspended into 200 µl of MPB by pipetting up and down. Flow cytometric analysis was performed with a BD Fortessa™ flow cytometer. All samples were mixed and acquired by the instrument, resulting in approximately 15,000–20,000 single bead events being recorded per sample. FlowJo software was used to analyze flow cytometric data. Median fluorescence intensity (MFI) for all 39 capture bead subsets were background-corrected by subtracting respective MFI values from the matched non-EV buffers that were treated exactly like EV-containing samples.

### Peripheral Blood Mononuclear Cells (PBMCs) Culturing

Isolated PBMCs were taken in each well in a 96-well plate. The first well was the control, which contained only the cells. The rest of the PBMCs were treated with 20 µg exosomes. The plate was kept in the incubator (37°C, 5% CO_2_) for 24 h for surface and intracellular staining, followed by flow cytometry. All the cells were cultured in RPMI 1640 mammalian cell culture medium (#72400-021, Gibco/Thermofisher) with 10% FBS (#A4736401, Thermofisher) and 1% of antibiotic/antimycotic (#15240096, Gibco/Thermofisher).

### Surface Staining for PBMC Characterization

Before starting the process of surface staining, the cells were treated with phorbol-12-myristat-13-acetate (PMA) (#P8139, Sigma-Aldrich), ionomycin (#I0634, Sigma-Aldrich), and brefeldin-A (#00-4,506-51, Thermofisher) (1:1:3; 1, 1, and 3 ug/ml). After 4-h incubation, an antibody cocktail containing (anti-human CD4-SuperBright 600 (#63-0047-42) and anti-human CD8-PerCP-eFlour 710 (#46-0087-42) (both from Thermofisher; described earlier (Singh et al., 2021)) diluted in DPBS was added to each well. Afterward, the plate was incubated in the dark for 30 min. After washing once, the plate was treated with 100 µl fixation solution (Fix/Perm buffer; #88-8824-00; Thermofisher) and stored at 4°C overnight.

### Intracellular Staining for Cytokine Measurements

The 96-well plate wastaken out from the 4°C storage for the intracellular staining procedure. 1x permeabilization buffer (#00-8333-56, Thermofisher) was used to wash the cells. The cells were resuspended into 50 µl of permeabilization buffer (1x) after 5-min centrifugation at 600 *g*. An antibody cocktail panel (all purchased from Thermofisher) (anti-human IFN-γ-Alexa Flour 488 (#53-7319-42), IL-17-PE-eFlour 610 (#61-7179-42), IL-2-APC (#17-7029-42), IL-10-PE-Cy7 (#25-7108-42), TNF-α-eFlour 450 (#48-7349-42), and CCL2/MCP-1-PE (#12-7096-82)) were diluted in 10 µl permeabilization buffer and 17.5 µl of the antibody cocktail was added into each well. After the addition of antibodies, the plate was incubated for 45 min. After washing once with DPBS, the cells were resuspended into 200 µl of DPBS to perform the flow cytometry. FlowJo software was used to analyze flow cytometric data.

### Statistics

Pregnant healthy control and COVID-19-recovered pregnant women were compared for their exosome levels using an unpaired t-test. For the comparison of the cytokines produced by either CD4^+^ or CD8^+^ T cells, the nonparametric one was analyzed using ANOVA and post hoc Tukey test. p-value considered significant if *p ≤ 0.05 and **p ≤ 0.01.

## Data Availability

The raw data supporting the conclusion of this article will be made available by the authors, without undue reservation.
